# Supplementary material for: Facile Preparation of Pd/UiO-66-v for the Conversion of Furfuryl Alcohol to Tetrahydrofurfuryl Alcohol under Mild Conditions in Water
Source: Nanomaterials (Basel). 2019 Nov 28;9(12):1698. doi: 10.3390/nano9121698 (PMC6956234; doi:10.3390/nano9121698)
Supplement: Supplementary file 1 [file nanomaterials-09-01698-s001.pdf]

Supplementary Information

Facile Preparation of Pd/UiO-66-v for the Conversion of Furfuryl Alcohol to Tetrahydrofurfuryl Alcohol under Mild Conditions in Water

Yanliang Yang [1,*], Dongsheng Deng [1], Dong Sui [1], Yanfu Xie [2], Dongmi Li [1] and Ying Duan [2,*]

[1] Henan Key Laboratory of Function-Oriented Porous Material, College of Chemistry and Chemical Engineering, Luoyang Normal University, Luoyang 471934, China; dengdongsheng168@sina.com (D.D.); suidonghy@mail.nankai.edu.cn (D.S.); lidongmi223@126.com (D.L.);

[2] College of Food and Drug, Luoyang Normal University, Luoyang 471934, China; xieyanfu1234@163.com (Y.X.)

* Correspondence: yangyli@mail.ustc.edu.cn (Y.Y.); duanying@mail.ustc.edu.cn (Y.D.); Tel.: +86-379-6861-8320 (Y.Y.); +86-379-6861-8516 (Y.D.)

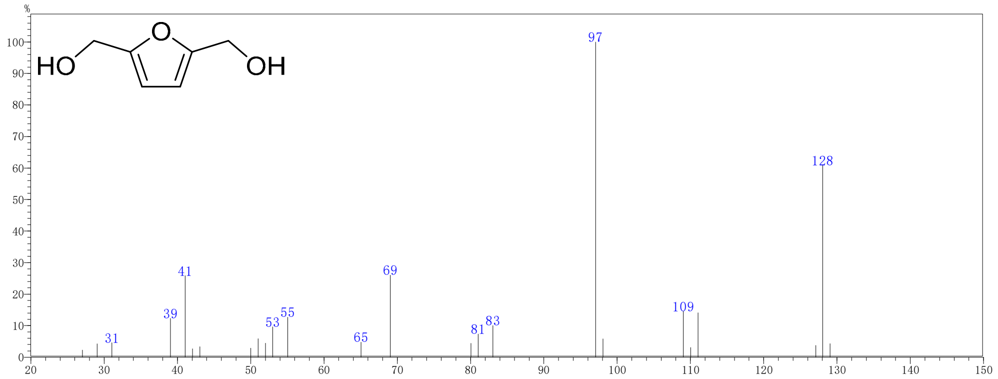

Figure S1. Mass spectrum for BHMF.

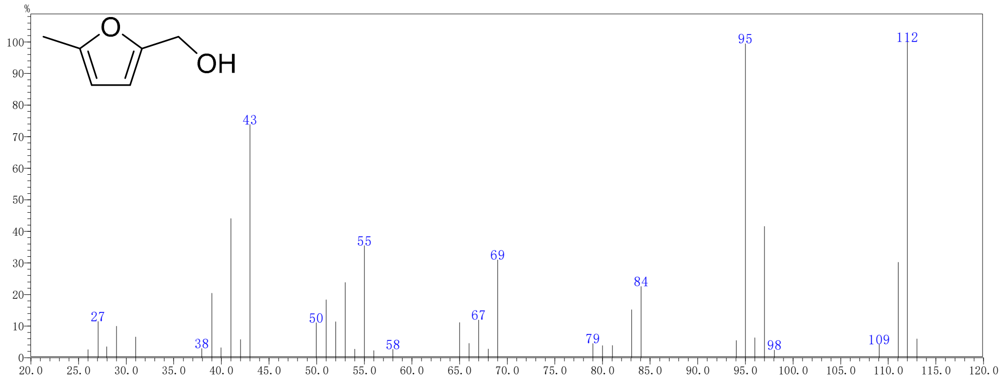

Figure S2. Mass spectrum for 5-methylfuran-2-methanol.

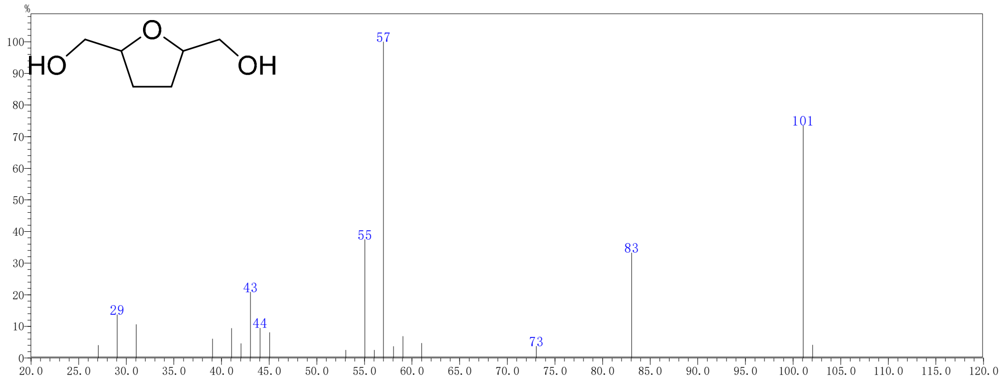

Figure S3. Mass spectrum for (tetrahydrofuran-2,5-diyl)dimethanol.

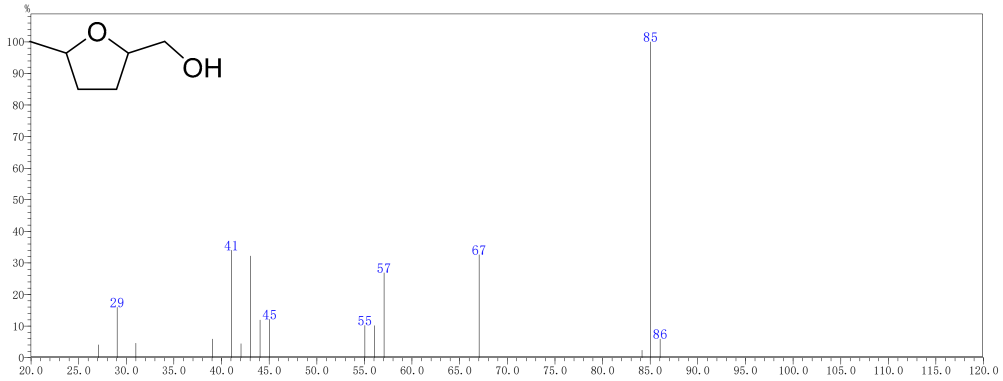

Figure S4. Mass spectrum for (5-methyltetrahydrofuran-2-yl)methanol.

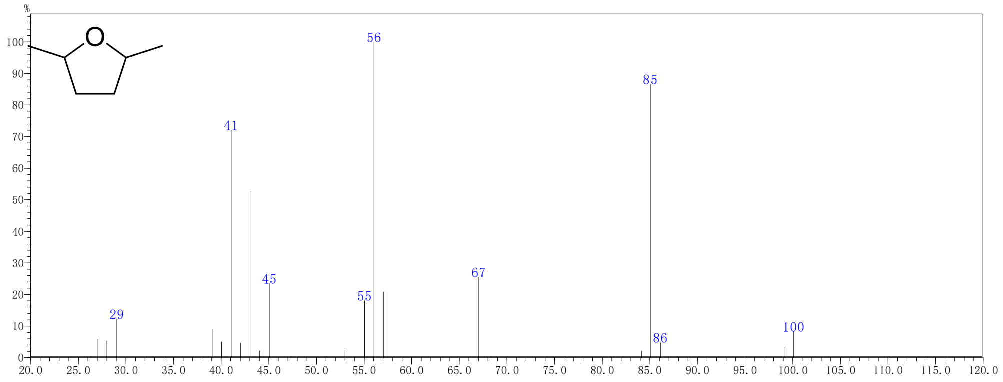

Figure S5. Mass spectrum for 2,5-dimethyltetrahydrofuran.

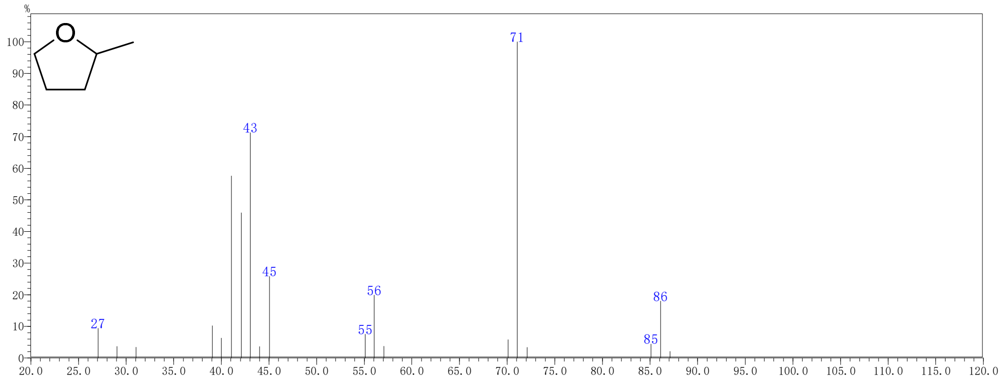

Figure S6. Mass spectrum for 2-methyltetrahydrofuran.

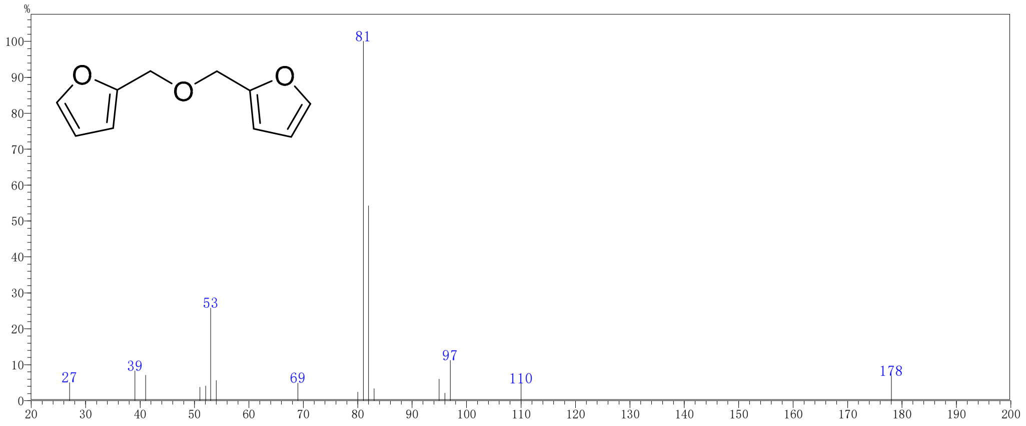

Figure S7. Mass spectrum for 2,2'-(oxybis(methylene))difuran.

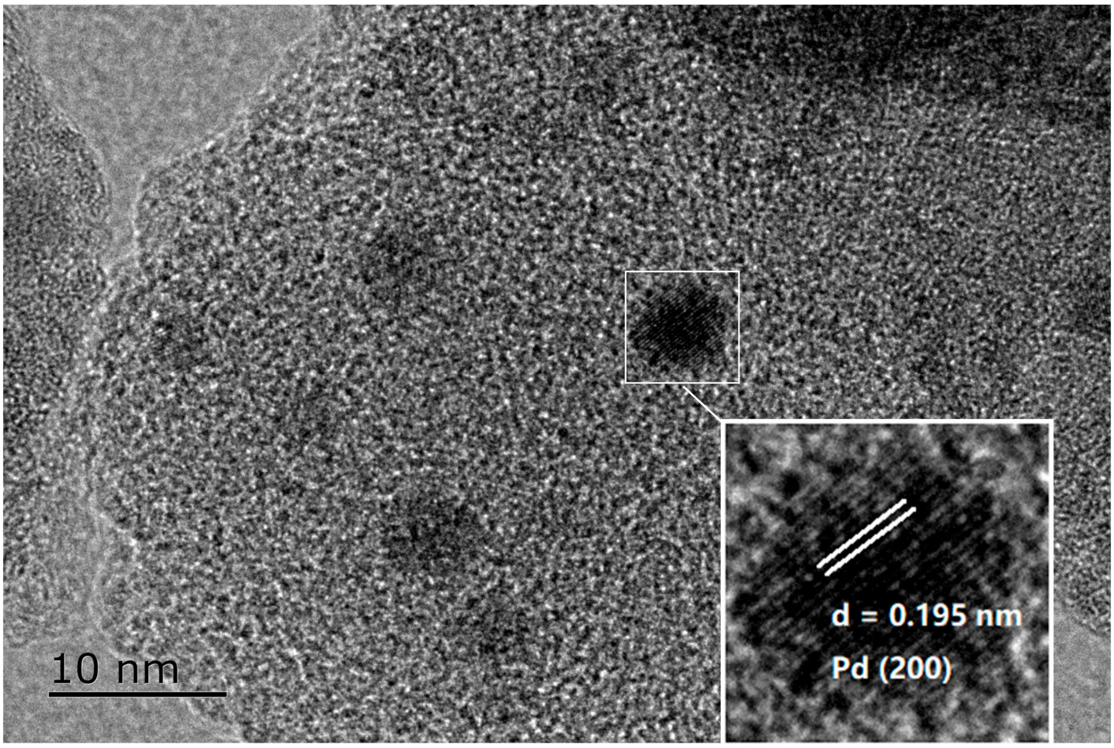

Figure S8 The HRTEM picture of Pd/UiO-66-v.

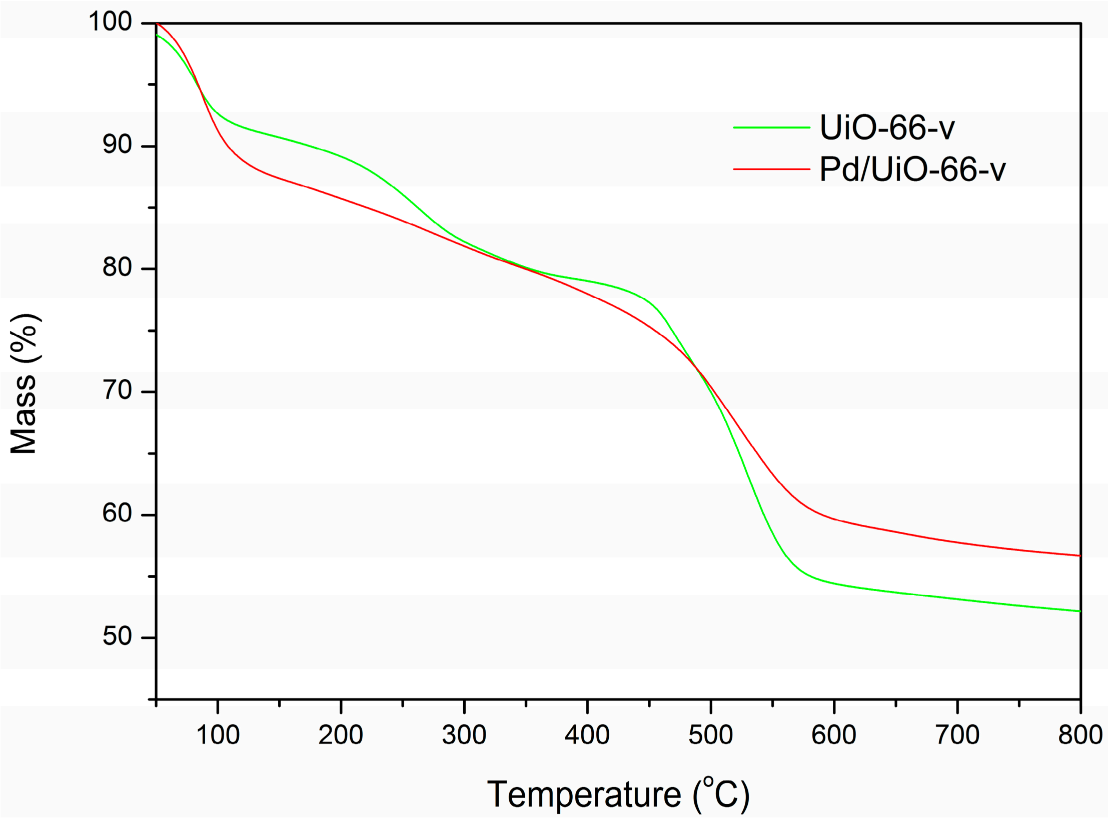

Figure S9. The TG curves for UiO-66-v and Pd/UiO-66-v.

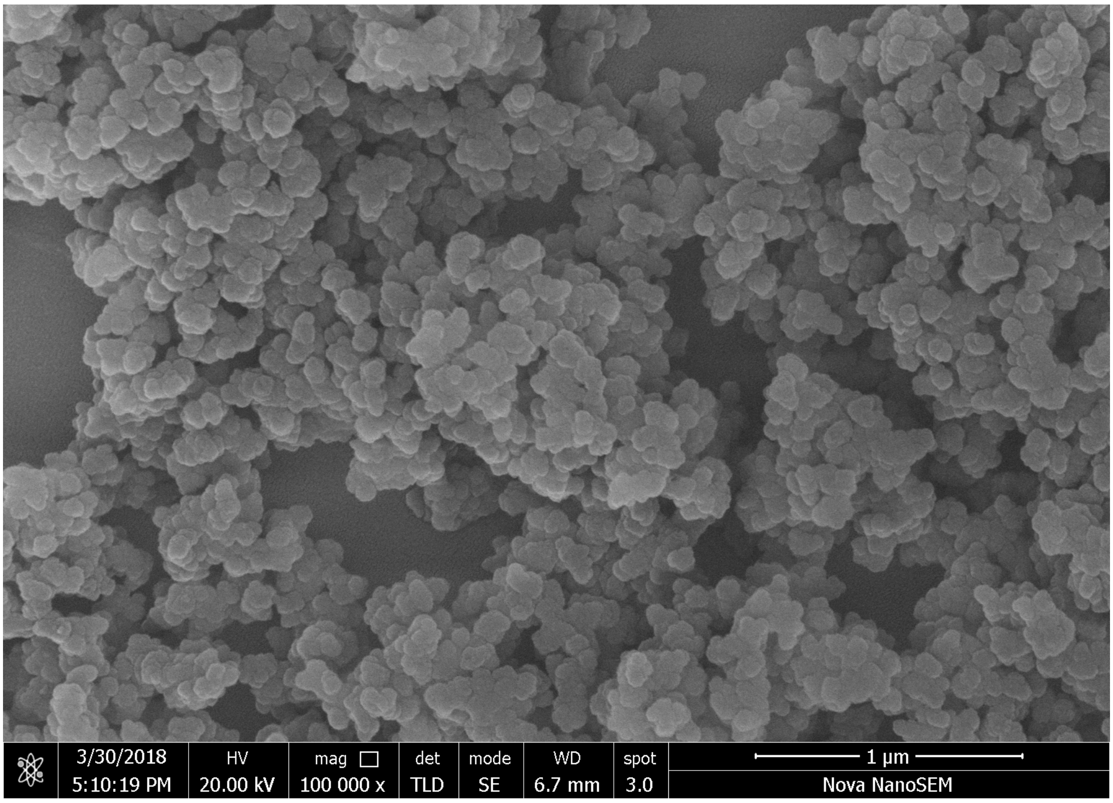

Figure S10. The SEM image for UiO-66-v.

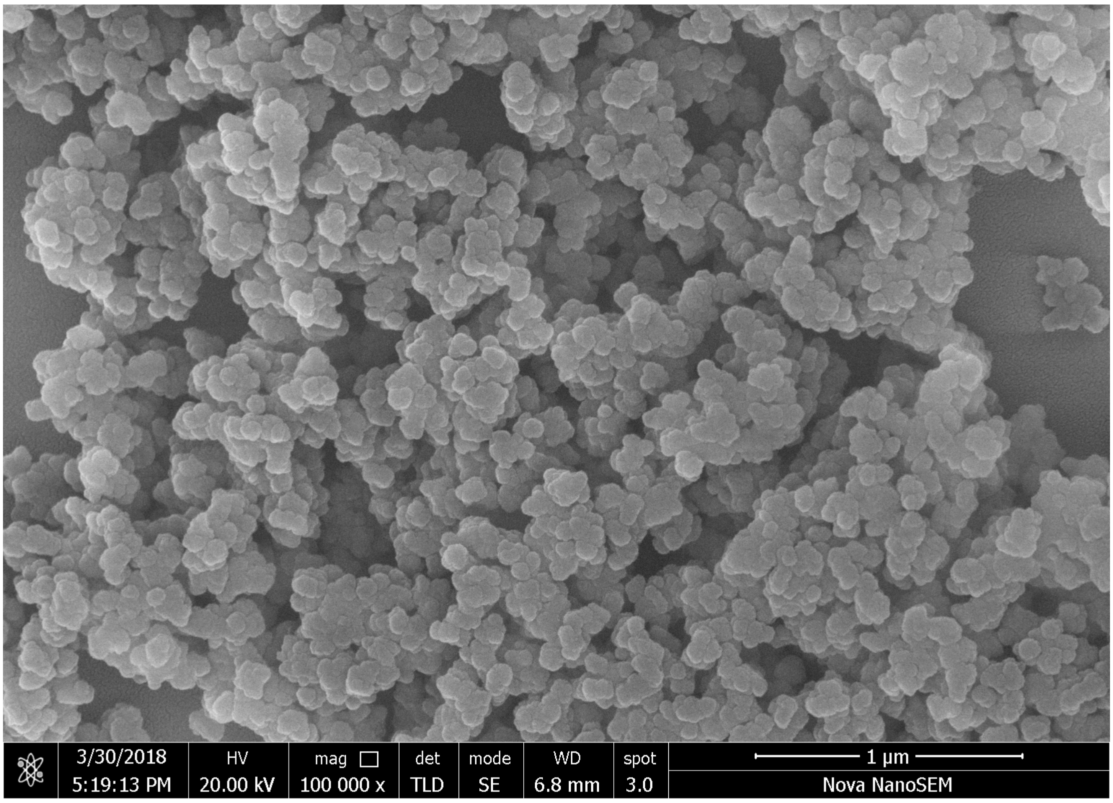

Figure S11. The SEM image for Pd/UiO-66-v.

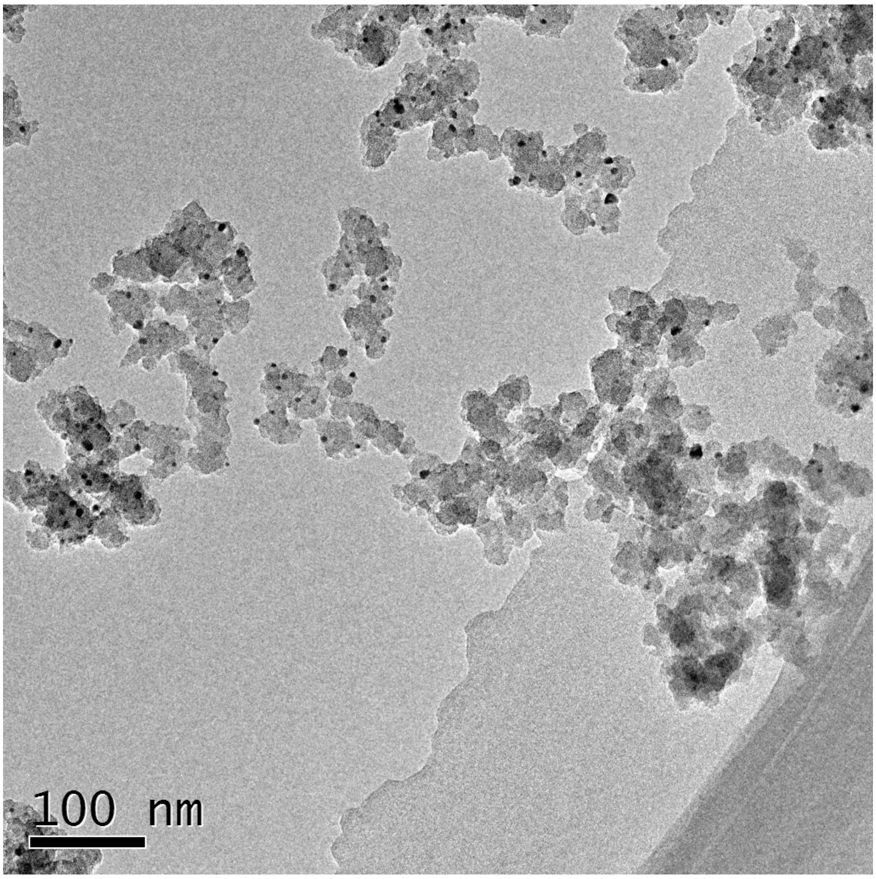

Figure S12. The TEM image for Pd/UiO-66-v.

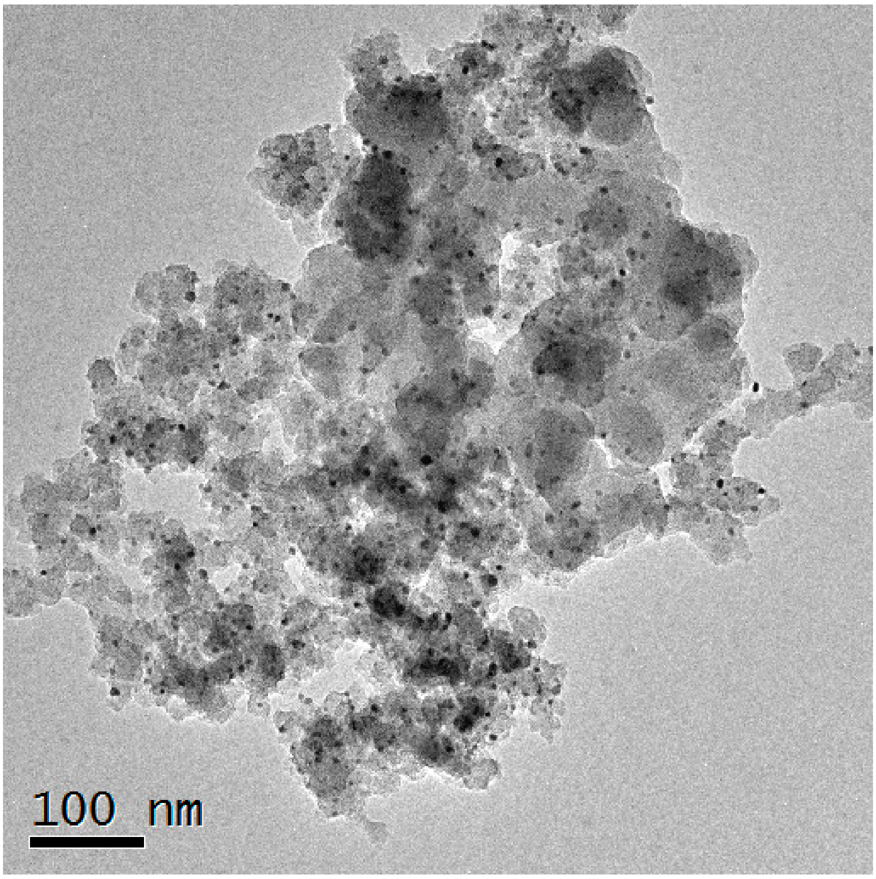

Figure S13. The TEM image for used Pd/UiO-66-v.

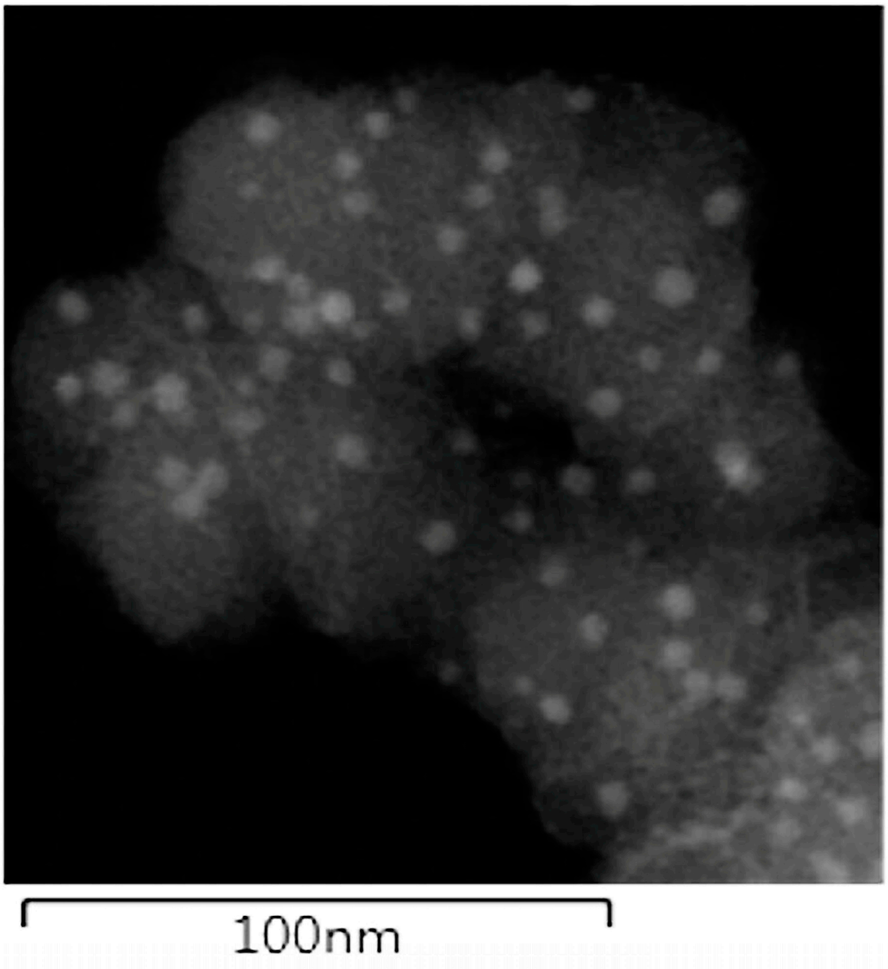

Figure S14. The electron image for Pd/UiO-66-v.

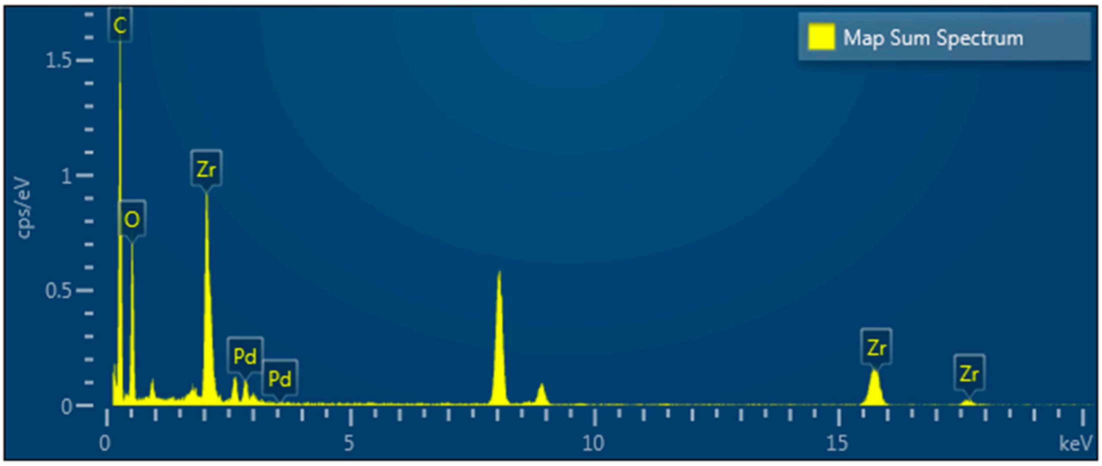

Figure S15. The map sum spectrum for Pd/UiO-66-v.

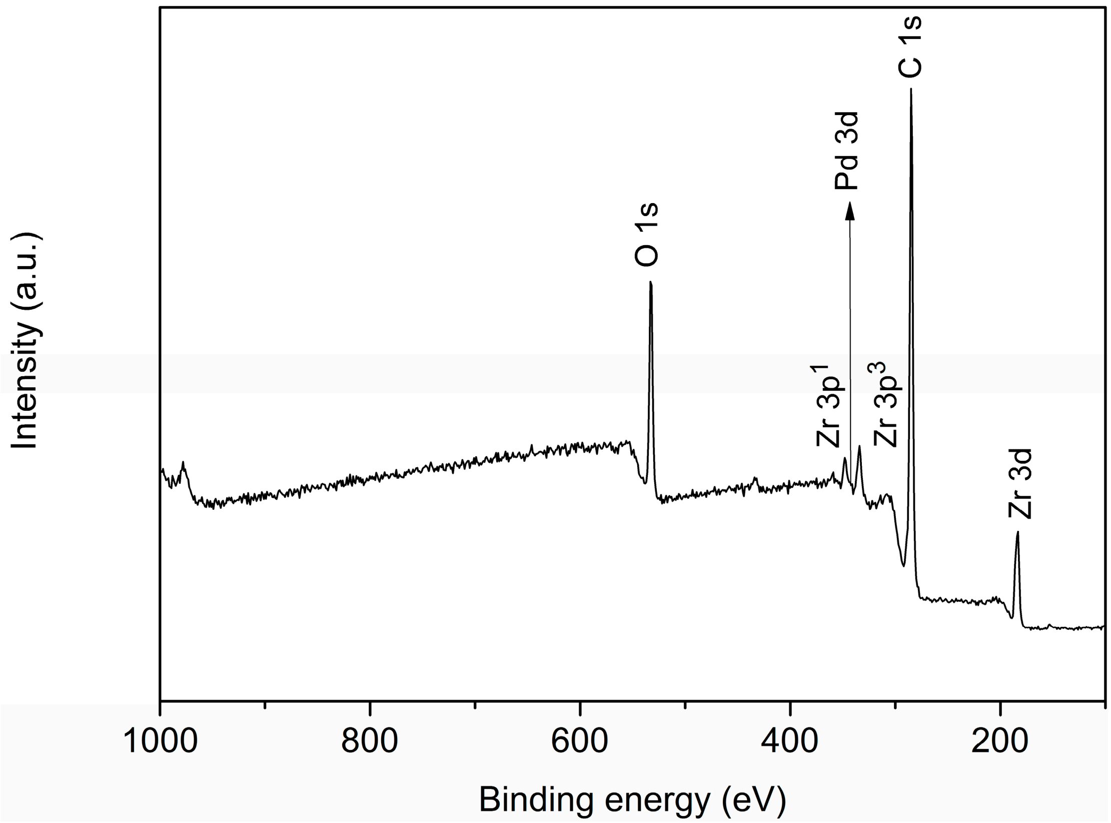

Figure S16. The survey XPS spectra of Pd/UiO-66-v.

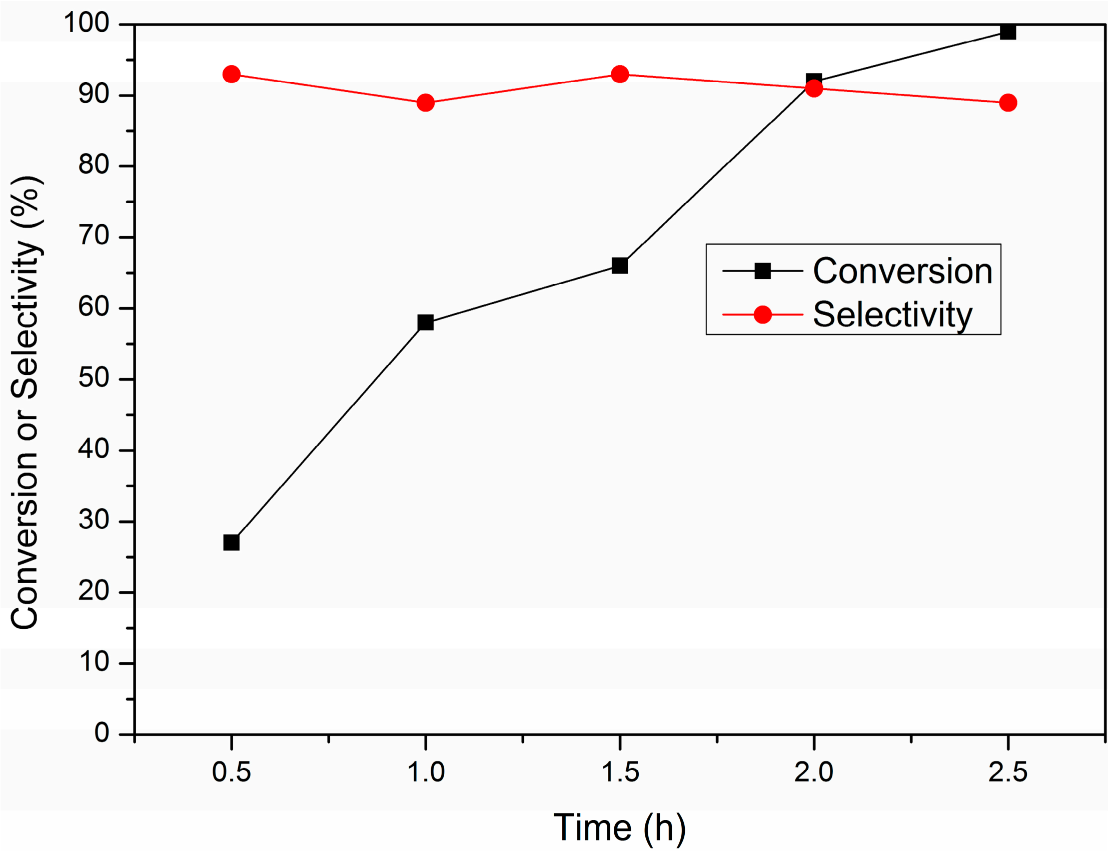

Figure S17. Influence of reaction time on the hydrogenation of furfuryl alcohol.
